# Antiferromagnetic coupling of TbPc_2_ molecules to ultrathin Ni and Co films

**DOI:** 10.3762/bjnano.4.36

**Published:** 2013-05-21

**Authors:** David Klar, Svetlana Klyatskaya, Andrea Candini, Bernhard Krumme, Kurt Kummer, Philippe Ohresser, Valdis Corradini, Valentina de Renzi, Roberto Biagi, Loic Joly, Jean-Paul Kappler, Umberto del Pennino, Marco Affronte, Heiko Wende, Mario Ruben

**Affiliations:** 1 Faculty of Physics and CENIDE, University Duisburg-Essen, Lotharstraße 1, 47048 Duisburg, Germany; 2 Institute of Nanotechnology, Karlsruhe Institute of Technology (KIT), 76344 Eggenstein-Leopoldshafen, Germany; 3 Centro S3, Istituto Nanoscienze - CNR, via Campi 213/a, I-41125 Modena, Italy; 4 ESRF, 6 rue Jules Horowitz, BP 220, F-38043 Grenoble Cedex, France; 5 Synchrotron SOLEIL, L′Orme des Merisiers, Saint-Aubin - BP 48, 91192 Gif-sur-Yvette, France; 6 Dipartimento di Scienze Fisiche, Informatiche e Matematiche, Università di Modena e Reggio Emilia, via Campi 213/A, I-41125 Modena, Italy; 7 Universite de Strasbourg, Institut de Physique et de Chimie des Materiaux de Strasbourg, CNRS UMP 7504, 23 Rue du Loess, 67034 Strasbourg Cedex 2, France

**Keywords:** magnetic anisotropy, magnetic coupling, single molecule magnets, X-ray absorption spectroscopy

## Abstract

The magnetic and electronic properties of single-molecule magnets are studied by X-ray absorption spectroscopy and X-ray magnetic circular dichroism. We study the magnetic coupling of ultrathin Co and Ni films that are epitaxially grown onto a Cu(100) substrate, to an in situ deposited submonolayer of TbPc_2_ molecules. Because of the element specificity of the X-ray absorption spectroscopy we are able to individually determine the field dependence of the magnetization of the Tb ions and the Ni or Co film. On both substrates the TbPc_2_ molecules couple antiferromagnetically to the ferromagnetic films, which is possibly due to a superexchange interaction via the phthalocyanine ligand that contacts the magnetic surface.

## Introduction

**Figure 1:**
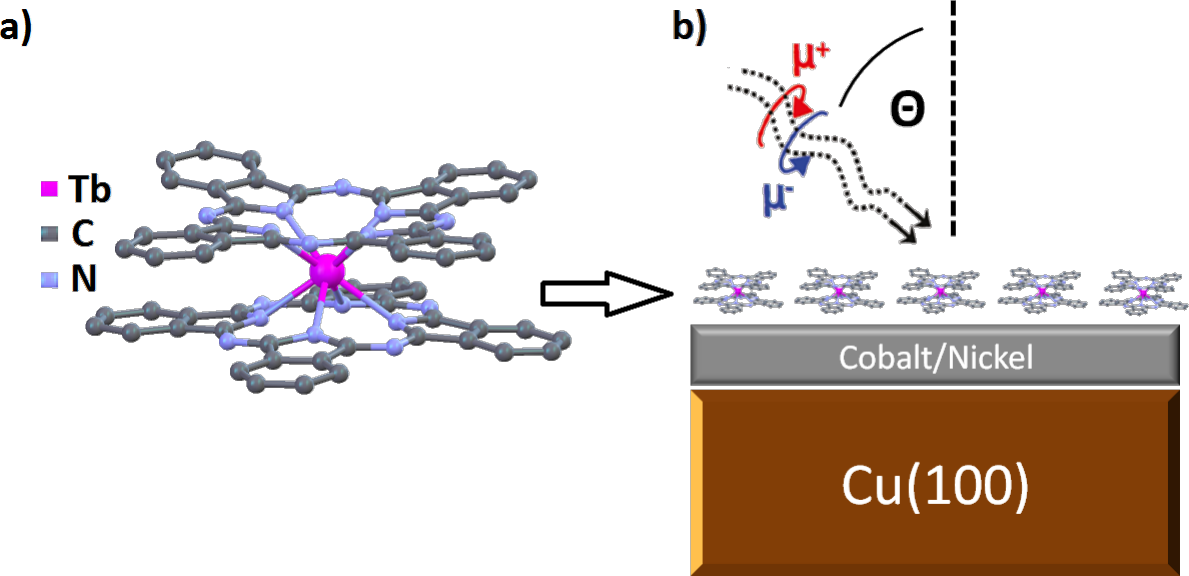
(a) The TbPc_2_ molecule, consisting of two parallel phthalocyanine planes with the Tb ion centred in between and (b) a schematic illustration of the sample studied.

Molecular spintronic devices as building blocks for future applications in information technology may be a big improvement and lead to higher efficiency [1–9]. Therefore several groups have studied the properties of potential organic molecules intensively over the past few years. Single-molecule magnets (SMMs) meet the requirements because of their magnetic remanence at low temperature without the need for long-range ordering. While most of the well-known SMMs consist of several interacting metal ions [10–13], bis(phthalocyaninato) terbium(III) (TbPc_2_) has only one rare-earth ion, coordinated by two organic phthalocyanine ligands consisting of nitrogen, carbon and hydrogen atoms (Figure 1a). The SMM properties arise simply from the single ion anisotropy of the Tb ion exhibiting a total angular momentum of *J* = 6 [14]. The crucial point with regards to the applicability of magnetic molecules for industrial usage is the control of the magnetic properties. Mn- and Fe-porphyrin molecules were successfully coupled to ferromagnetic thin films, leading to the alignment of the magnetic moments of the metal ions parallel to the film [15–17] or antiparallel due to an interlayer of oxygen [18–19]. Recently, it was shown that TbPc_2_ molecules can be magnetically coupled to a ferromagnetic Ni substrate [20]. The magnetic anisotropy and field dependence were also studied for TbPc_2_ in a submonolayer on Cu(100) [21] and on antiferromagnetic substrates [22]. Spin quantum tunnelling at zero magnetic field leads to vanishing magnetization of the molecules, showing typical butterfly hysteresis [23–25]. Only at very low temperatures below 4 K is the relaxation slow enough for observation of magnetic remanence [26]. Here we demonstrate that it is possible to block this relaxation and conserve the magnetization without any external field by coupling the molecules to a ferromagnetic substrate. By X-ray absorption spectroscopy and X-ray magnetic circular dichroism we study the electronic and magnetic properties of a submonolayer coverage of TbPc_2_ molecules on ultrathin Co and Ni films, focusing on the magnetic coupling between the substrate and the molecules.

## Results and Discussion

### X-ray absorption spectroscopy of TbPc_2_ molecules

**Figure 2:**
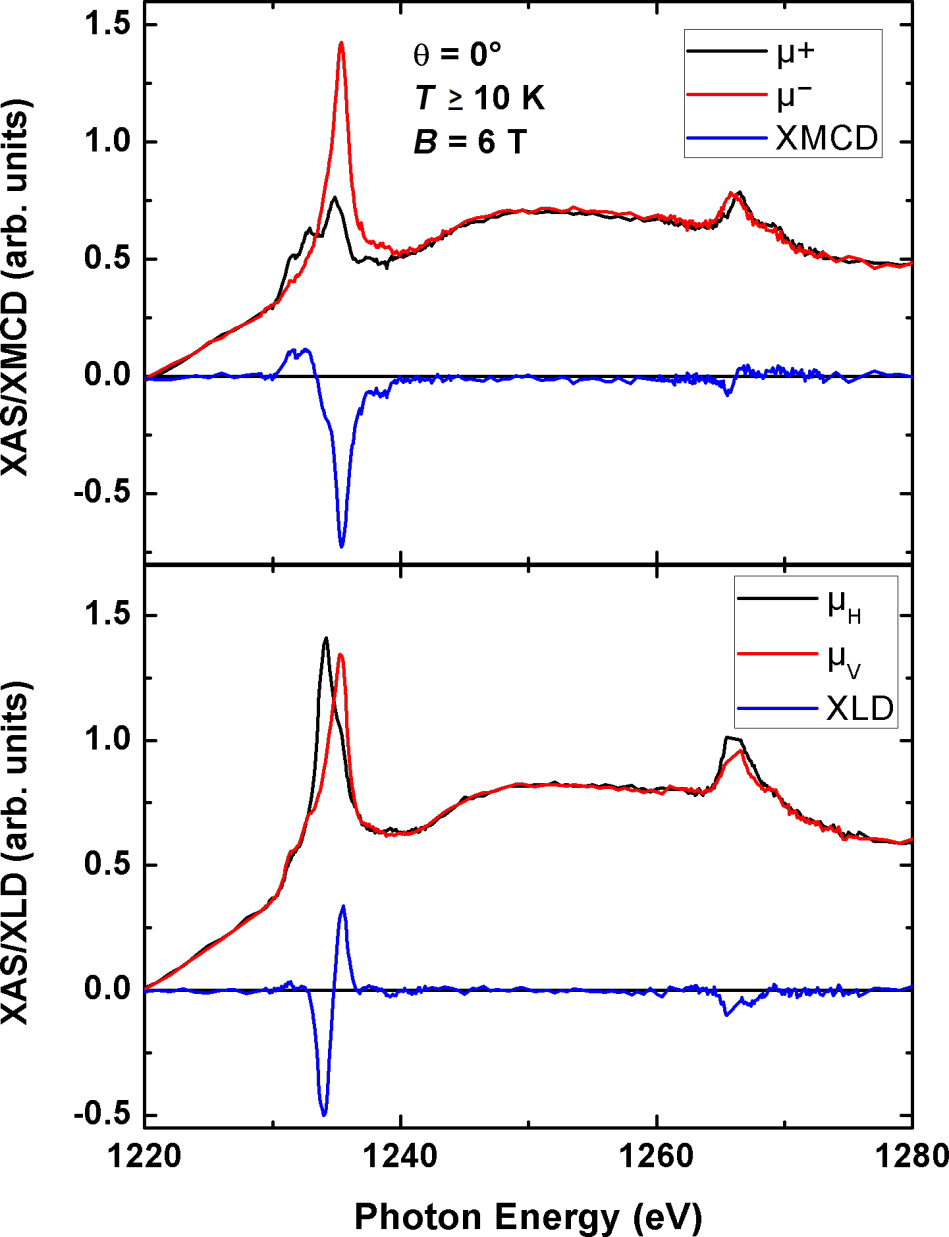
X-ray absorption spectra of the Tb M_4,5_ edges for a submonolayer TbPc_2_/Cu(100), measured at *T* ≥ 10 K and *B* = 6 T. The top shows the spectra for circularly polarized X-rays (black and red) and the µ^+^ − µ^−^ = XMCD signal (blue) at perpendicular photon incidence (0°). The bottom figure shows the linearly polarized absorption spectra for an incidence angle of 45°. The red line corresponds to vertical polarization, where the electric field vector is parallel to the sample plane. The black line corresponds to horizontal polarization with an angle of 45° between the electric field vector and the sample surface. The blue line is the difference, i.e., the µ_V_ − µ_H_ = XLD signal.

Figure 2 shows spectra of circularly polarized (top) and linearly polarized X-rays (bottom) and the corresponding circular or linear dichroism at the Tb M_4,5_ absorption edges of TbPc_2_ on Cu(100). The XMCD signal has the shape typical for a Tb^3+^ ion, in agreement with what has been already reported for this molecule [20–21,24–25]. The high XMCD intensity at the M_5_ edge and the low intensity at the M_4_ edge indicate the large orbital moment of the Tb ion [27–28]. From the X-ray linear dichroism (XLD) at 45° X-ray incidence, one can deduce the orientation of the molecules. The typical shape at the M_5_ edge, with the sign changing from negative to positive, indicates that the molecules lie flat, with the phthalocyanine planes parallel to the surface, as expected for the submonolayer coverage [25].

### Magnetic coupling on a Ni surface

**Figure 3:**
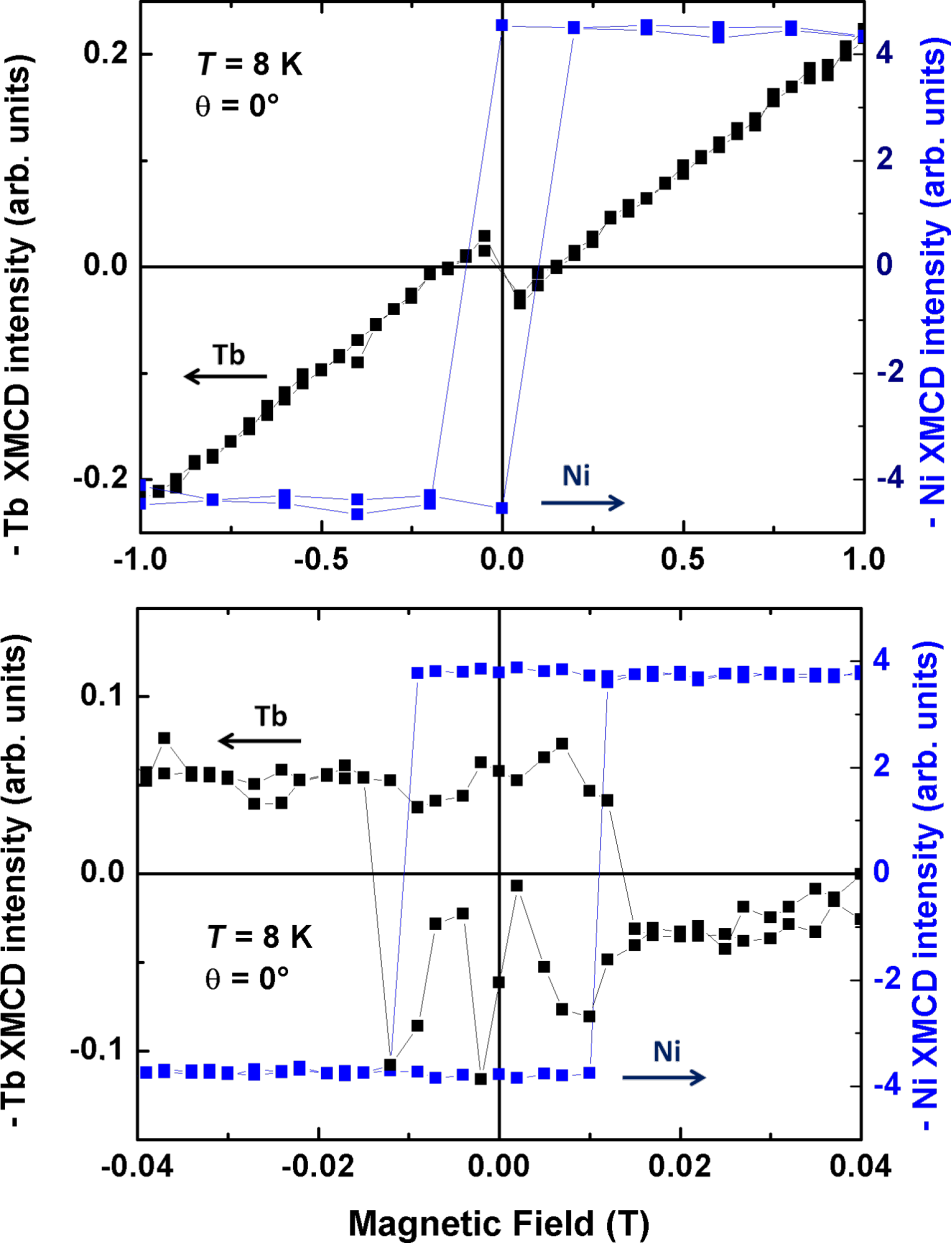
Element-specific field-dependent magnetization for the sample TbPc_2_/Ni/Cu(100). The blue line is the magnetization curve at the maximum XMCD intensity of the Ni L_3_ edge (852.5 eV), the black line is at the Tb M_5_ edge (1235.4 eV). The top figure shows the curves from −1 T to 1 T and the bottom figure presents a zoom between −0.04 T and 0.04 T. The measurements were performed at *T* = 8 K under normal X-ray incidence (0°).

The 15 ML thick Ni film has a well-defined easy magnetic axis perpendicular to the plane [29] and the TbPc_2_ molecule is known for its large magnetic anisotropy with the easy axis of magnetization perpendicular to the phthalocyanine plane [21]. Thus, the easy axis of the molecules is parallel to the one of the underlying Ni film. From the field-dependent XMCD at both Tb M_5_ and Ni L_3_ absorption edges presented in Figure 3, the influence of the Ni film on the magnetization of the molecules is visible. While the magnetic moments of Tb align parallel to the external magnetic field when the field is strong enough, they align antiparallel in a small magnetic field. This antiparallel alignment is caused by the magnetic coupling to the Ni substrate. At the top of Figure 3 one is able to identify the coupling by the magnetization sign changes. The signal crosses zero three times, twice when the exchange field and the external field cancel out each other and once when the Ni magnetization crosses zero. In the image below the coupling is even more visible. In this very small region around zero, one can see the remanence of the Ni substrate. For the Tb signal there is remanence as well, but with the opposite sign. The magnetization direction for Ni and Tb switch exactly at the same external field of about ±0.01 T, demonstrating the interaction between the molecules and the Ni film. Please note that the connecting lines for the Ni data in the upper picture of Figure 3 do not represent the Ni hysteresis curve, because of the low density of magnetic field steps. A more precise representation of the Ni hysteresis and the corresponding coercive field can be seen in the lower figure. Since the identical system was investigated by Lodi Rizzini et al. [20], it is remarkable that we obtain a significant difference in the exchange field and the amplitude of the antiparallel signal at the Tb M_5_ edge, probably as a result of a weaker coupling. However, the main issue of the present manuscript is the demonstration of an antiferromagnetic coupling of rare-earth–Pc_2_ molecules to ferromagnetic substrates. The quantitative analysis of the antiferromagnetic coupling strength goes beyond the scope of this manuscript. This can be achieved by experimental investigation by means of a detailed temperature-dependent XMCD study together with comparison to ab initio calculations, e.g., by utilizing density functional theory (DFT).

### Magnetic coupling on a Co substrate

**Figure 4:**
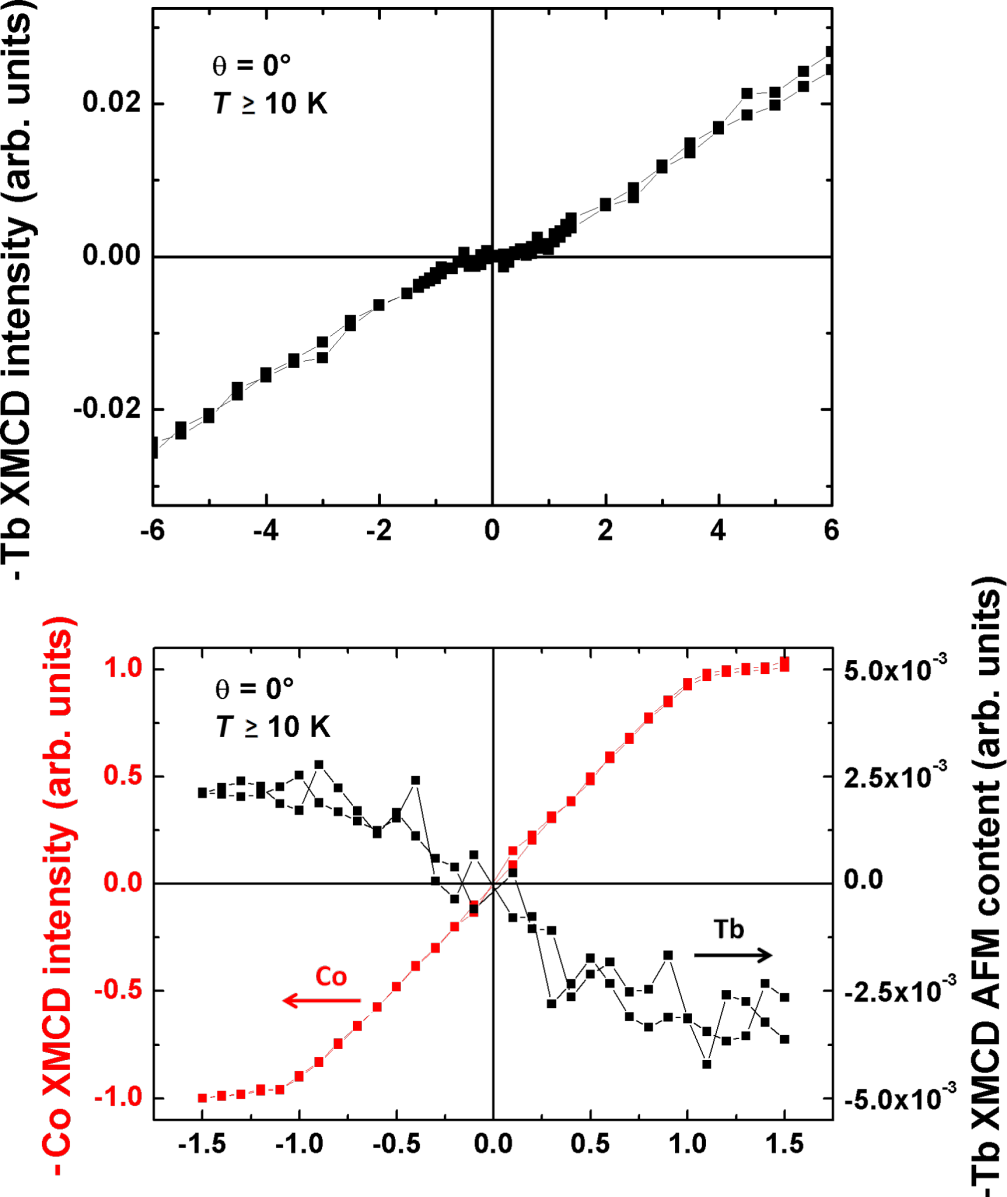
Magnetization curves for the sample TbPc_2_/Ni/Cu(100). The top figure shows the magnetization curve at the maximum of the M_5_ edge of Tb (1235.4 eV) from −6 T to 6 T. In the bottom figure one can see the magnetization curve at the maximum of the L_3_ edge of the Co XMCD signal (778 eV) (red line) and the Tb M_5_ curve after subtraction of a linear signal (black curve). The measurements were performed at perpendicular X-ray incidence at *T* ≥ 10 K.

The important difference between the Co and the Ni substrate is the orientation of the magnetic easy axis that is parallel to the surface for the 10 ML thick Co film [30]. The magnetic coupling between the Co substrate and the molecule is therefore only between the out-of-plane component of the Co magnetization. The parallel component cannot polarize the molecules because of their large magnetic anisotropy [20]. The top graph of Figure 4 shows the magnetic signal of Tb in an external field between −6 T and 6 T. The shape is dominated by a paramagnetic signal that one would expect for a free molecule at a temperature of *T* ≈ 10 K. But close to zero field the superposition of a second contribution becomes visible. In order to highlight the contribution, we subtracted the magnetization signal expected for the free molecule, which is simply a linear background at this temperature. The outcome is the lower graph of Figure 4. Compared to the Co magnetization curve (red line), one can see the antiparallel signal of the Tb magnetization. This antiferromagnetic contribution is very small compared to the dominating paramagnetic signal, because the Co magnetization direction is not in the direction of the molecular easy axis. If a very large coupling strength between the molecules and the Co substrate exceeds the magnetic anisotropy barrier, the magnetic moment of Tb could be forced in the plane. However, because of the large magnetic anisotropy barrier of the TbPc_2_ molecules of 73 meV [20,31], we expect the magnetic moment of the Tb ions not to be forced to the in-plane direction by the Co magnetization since the coupling energies presented in [20] are in the regime of 1 meV. Nevertheless, this needs to be demonstrated, e.g., by angle-dependent measurements. In addition, the calculated coupling strength compared to the anisotropy energy would answer this question. But as mentioned above, this goes beyond the scope of this manuscript. Another reason for a smaller antiferromagnetic coupling strength may be the higher temperature for the measurements on Co (T ≥ 10 K) than on Ni (8 K). This temperature difference may cause changes in the field dependence of the XMCD, because a significant temperature dependence of the magnetic coupling is expected [20].

## Conclusion

We have shown that the magnetic moments of TbPc_2_ molecules can be ordered by coupling to a ferromagnetic substrate. At a temperature of 8 K it is possible to get a remanent magnetization, originating from an antiferromagnetic coupling to the Ni substrate. We observe an antiferromagnetic coupling also on the Co substrate, whose magnetic easy axis is in the plane and thus perpendicular to the one of the terbium(III) total angular momentum of *J* = 6. This antiferromagnetic contribution is much smaller than on the Ni film, partially due to the higher measuring temperature, but primarily because of the large magnetic anisotropy of the molecule. Therefore the magnetization curve is dominated by the paramagnetic signal.

## Experimental

The samples were prepared in situ at a base pressure of 10^−10^ mbar directly before the measurements. By cycles of Ar^+^ bombardment and annealing the Cu(100) surface was cleaned. Afterwards the ferromagnetic films were grown epitaxially via electron beam evaporation. The 10 ML thick Co film was produced with a rate of 0.5 ML/min and the 15 ML thick Ni film with a rate of 0.3 ML/min. The TbPc_2_ molecules were thermally evaporated at a temperature of about 400 °C from a Knudsen cell onto the substrate held at room temperature.

The XAS measurements of the system TbPc_2_/Co/Cu(100) were performed in magnetic fields up to 6 T at the DEIMOS beamline at the synchrotron SOLEIL, the XAS measurements of the system TbPc_2_/Ni/Cu(100) in a magnetic field up to 4 T at the ID08 beamline at the ESRF. The lowest possible temperature was reached by cooling with liquid helium (*T* = 4 K), while the real temperature at the sample was limited by the quality of the thermal contact, leading to temperatures above 4 K. In detail we were able to measure at *T* ≈ 8 K at the ESRF and *T* ≥ 10 K at SOLEIL, although the temperature at the sample holder was 4 K.

To determine the thermal stability of TbPc_2_, the sample was exposed to high-temperature sublimation under high vacuum in the sublimation apparatus consisting of a tube flask dipped into a heating mantle and a condenser. This method is carried out by heating 80 mg of the crude solid, while simultaneously evacuating the system. Upon reaching 400–420 °C at the bottom of the round flask and at a standard pressure in the system of about 2.8·10^−2^ mbar, the sublimate appears on the cooled condenser. After 40 min about half of the crude solid was collected on the condenser. Both the sublimate and the residual molecules in the tube flask were exposed to mass-spectrometric and spectroscopic analysis as was the rest of the sample from a Knudsen cell. MALDI-TOF and UV–vis spectra did not reveal any difference compared to the initial sample. The presence of a binuclear complex (Tb_2_Pc_3_) in the MALDI-TOF spectrum as an evidence of thermal degradation was detected only in the rest of the sample in the tube flask after exposure to more than 450 °C for not less than 30 min. This allows us to assert that sublimation at 400 °C leaves the molecules unaffected.
